# Chicago Medical Society

**Published:** 1866-11

**Authors:** 


### PROCEEDINGS OF MEDICAL SOCIETIES.

### CHICAGO MEDICAL SOCIETY.

Dr. Reid read an excellent report of a case of placenta prævia, which may be found in full on another page.

### ATOMIZING TUBES.

Several members testified to the great benefit which patients, suffering from catarrhal affections not only of the nasal passages and fauces but also of the lungs, had derived from the inhalation of astringent solutions thrown from atomizing tubes in the form of spray.

### CONCUSSION FOLLOWED BY JAUNDICE.

Dr. Bevan reported a case of concussion, in which, for thirty-six hours, there was great degree of coma. At the end of this period bloody serous fluid escaped from the ear. On the fourth day the patient became jaundiced, the conjunctiva and skin being deeply discolored.

Dr. Fisher had seen several cases of jaundice following concussion, and regarded the symptom as one of great gravity.

### SUPPRESSION OF URINE IN CHOLERA.

Several cases were reported in which suppression of urine was a most serious complication of cholera. A case, reported by Dr. Reid, is of particular interest. A young man, previously in perfect health, had been relieved of excessive vomiting, choleraic diarrhœa, severe cramps and collapse, after the use of calomel and minute doses of opium. Reaction was re-established and bilious discharges had returned, when symptoms of suppression of the urine appeared. No urine was passed voluntarily nor could be drawn by the catheter for three days; and yet the patient was perfectly conscious, although exceedingly restless and agitated. Diuretics were freely administered; minute doses of morphia became necessary to give quietness. At the end of the third day, great prostration occurred. Although no urine could be drawn from the bladder, consciousness remained almost to the very last. Death occurred on the fourth day from the commencement of the suppression.

### TYPHOID FEVER.

The appointed subject of discussion for the evening was the treatment of typhoid fever.

There was a long and interesting discussion, in which nearly all the members took part, but for which we have no space. It may be worthy of notice, however, that Dr. O. Smith and others, who had enjoyed a long experience in Eastern States, called the attention of the Society to the fact, that typhoid fever at the East was a much more serious disease than here; that the disease required very little active treatment either at the East or West, and yet at the East patients would tolerate more active treatment than here.

### REPORT OF COMMITTEE ON THE NEW ANÆSTHETIC AGENT—NARCOTINA.

Dr. Bogue, Chairman of the Committee appointed to investigate the merits of a new anæsthetic, presented to the Society a few weeks since by Dr. Gilman, reported as follows:

He had witnessed the administration of the preparation to three patients requiring operations at the Cook County Hospital.

1st. A strong, healthy man, 35 years of age, inhaled the agent seventeen minutes with scarcely any effect either upon the respiration, pulse, consciousness, or sensibility. Chloroform was required for the operation.

2d. A man, similar in appearance with the above, inhaled the agent sixteen minutes with very little effect. In both these cases Dr. Gilman superintended the administration in person, and stated that continued inhalation would probably not produce anæsthesia.

3d. The preparation was administered to a little girl, 5 years of age, for five minutes, when perfect anæsthesia was produced. In this case the immediate and subsequent effects upon the patient were very satisfactory.

Several days after these experiments, Dr. Gilman again administered another preparation, said to be stronger than the first, to the first patient mentioned above. No opportunity was given the Committee of examining this preparation. In six minutes the patient was fully anæsthetized. During the next twenty-four hours, he was affected with disagreeable nausea.

On motion of Dr. Durham, it was

*Resolved*, That, after an examination and fair trial by the Committee of the preparation presented to the Society under the name of Narcotina, this Society has no confidence in it as an anæsthetic.

PUNCTURED WOUND OF FŒTUS IN UTERO.

Dr. Bogue exhibited a fœtus apparently of the third month, which had been expelled from a young married woman, after the introduction of a "probe" into the uterus, by a practitioner of this city. Behind and above the right clavicle was a minute punctured wound, as if produced by the probe. Examination showed that this opening extended into the cavity of the chest.

INTERNAL USE OF CHLOROFORM.

Dr. Bogue described the effects of a teaspoonful dose of chloroform, administered in sweetened water, to a strong Irishman, for severe colic pain in the abdomen, which five doses of morphine (gr. $\frac{1}{8}$) every two hours failed to give permanent relief.

Immediately after taking the chloroform, the patient suffered a severe pain in the stomach for half a minute, when he commenced panting violently, laughing and talking wildly. He then lay upon the bed, continuing to laugh and talk about three minutes; at the expiration of five minutes more, he was fully anæsthetized. For about fifteen minutes his breathing was slow and stertorous; his pulse descending from 80 to 48 beats per minute; the veins turgid, lips and face purple.

Sinapisms were applied to the abdomen and heat to the feet. The pulse and respiration became quite normal in a few moments. Slight vomiting occurred, when the patient slept quietly nearly an hour and a half. On awaking, he remained entirely free from pain.

Several members gave most favorable reports from experience regarding the use of chloroform, in doses varying from fifteen drops to a teaspoonful, in water, every two or three hours, for nausea and for pain (colic) in the abdomen.

